# Simulated carbon K edge spectral database of organic molecules

**DOI:** 10.1038/s41597-022-01303-8

**Published:** 2022-05-16

**Authors:** Kiyou Shibata, Kakeru Kikumasa, Shin Kiyohara, Teruyasu Mizoguchi

**Affiliations:** 1 Institute of Industrial Science, the University of Tokyo, 4-6-1, Komaba, Meguro-ku, Tokyo, 153-8505 Japan; 2 Laboratory for Materials and Structures, Institute of Innovative Research, Tokyo Institute of Technology, Yokohama, 226-8503 Japan

**Keywords:** Electronic structure, Cheminformatics

## Abstract

Here we provide a database of simulated carbon K (C-K) edge core loss spectra of 117,340 symmetrically unique sites in 22,155 molecules with no more than eight non-hydrogen atoms (C, O, N, and F). Our database contains C-K edge spectra of each carbon site and those of molecules along with their excitation energies. Our database is useful for analyzing experimental spectrum and conducting spectrum informatics on organic materials.

## Abstract (summary)

**Table:** 

Measurement(s)	electron energy loss spectroscopy
Technology Type(s)	density functional theory calculation
Factor Type(s)	organic molecules • carbon sites in a organic molecule

## Background & Summary

Carbon-based organic molecules form an immense configuration space and still have a lot of potentials for unknown functionalities in various fields. In the research and development of organic materials and their functionalities, accurate characterization of configuration and prediction of functionalities are the keys for success.

Among analytical clues for materials characterization, core-loss spectra, electron-energy loss near edge structure (ELNES) and X-ray absorption near edge structure (XANES), have been widely used as one of the most effective fingerprints for determination of local atomic structures and electronic states. These core-loss spectroscopy measure energy loss due to excitation of an electron from a core orbital and the core-loss spectra contain a partial density of states of the unoccupied states and possess useful information on atomic structure and electronic structure. Analysis of the core-loss spectrum have been performed by a comparison of measured spectra to reference spectra. Recent developments in experimental equipment and facilities have enabled core loss spectroscopy at high resolution regarding time, space, and energy. On the other hand, because of the extremely large amount of spectral data obtained, it is becoming increasingly important to establish methods for efficient and automatic analysis. Organic molecules have a variety of molecular structures, and the correlation between their structures and chemical bonds and core-loss spectral shapes is complex and has been remaining elusive.

These situations have been stimulating the development of reference spectral databases including huge variety of spectra obtained by experiments^1^ and calculation^2^. Although a database of the core-loss spectra for inorganic materials has been recently developed^3^, databases of the organic molecules are highly limited. Furthermore, in the field of materials science, application of informatics on predicting and designing various materials properties from spectrum data in data-driven manner has been attracting much interest. These facts represent increasing need for spectrum database of organic materials.

Here, we calculated core-loss spectra of carbon K (C-K) edge of 117,340 symmetrically unique sites in 22,155 molecules in a structure and property database of organic molecules^4,5^, based on density functional theory (DFT). Our dataset provides theoretical fingerprints for analyzing experimental core-loss spectra, and also offers an opportunity for trying data-driven spectrum informatics on organic molecules.

## Methods

### Density functional theory calculations

The calculations of C-K edge spectra and excitation energies were carried out based on DFT by the first-principles plane-wave basis pseudopotential method using CASTEP code^6–11^. The generalized gradient approximation in a Perdew-Burke-Ernzerhof (GGA-PBE)^12^ was adopted for the exchange-correlation functional. Spin polarization was not considered. For each carbon site in each molecule, an excited electronic structure was separately calculated using an on-the-fly potential of carbon. In the pseudopotential calculation, the core-hole effect can be taken into account by employing a special pseudopotential designed for the excited atom with a core-hole^11^. We consider a neutral excited state including a full core hole, *i.e*. a state with a 1 s core hole and an additional electron at an orbital corresponding to the original lowest unoccupied molecular orbital (LUMO) state, to calculate both the excitation energy and the spectral feature. The absolute value of the excitation energy was calculated as the total energy difference between the ground state and the excited state, so called Δ self-consistent field (Δ SCF) method^13^. The calculating the excitation energy in the Δ SCF scheme has been applied not only for all electron calculations but also for the pseudopotential calculations with PAW method by correcting contribution of the core orbitals to energy^14,15^. The spectral feature whose energy is relative to the energy of LUMO level was calculated as the pair of the dipole transition matrix elements between the core states and the unoccupied eigenstates. The peak positions relative to the transition to LUMO state are obtained as the difference in eigenvalues between the LUMO state and the unoccupied eigenstates. Using this method, not only the spectral features but also the chemical shift of the spectrum can be calculated. The cut-off energy of the plane wave 500 eV was used for all calculations. For the calculation of spectrum, 1,000 unoccupied levels were considered for all molecules.

We have selected the calculation method for the present study because of the following reasons: (1) The pseudopotential method is more efficient than the all-electron method because core-orbitals are not explicitly calculated^9^. (2) The CASTEP code adopts a pseudopotential method which provides more accurate theoretical spectra than those obtained by a muffin-tin potential method especially for systems with anisotropic covalent bondings^16^. (3) The calculation method has been applied for the core-loss spectra of many kinds of materials, including crystalline, doped graphene, liquid, and gaseous materials^14,17–23^.

Limitations and inaccuracies of this method can be summarized as the following: By setting the limited number of unoccupied levels, the spectra corresponding to the transitions to 1,000 unoccupied eigenstates are obtained. In the case of the carbon K-edge of the molecule calculated here, this corresponds to an energy range of up to 25 eV relative to the LUMO level, although it varies depending on the molecule and site. Using the pseudopotential method can save calculation costs, but it needs correction of energy contribution from the core orbitals that are not explicitly calculated. It has been reported that the excitation energy obtained by Δ SCF may vary up to about 20 eV depending on target elements, the calculation basis function set, potential description, and their practical implementations in calculation code^18^. The treatment of the exchange-correlation functional and DFT formalism also affects the evaluation of excitation energy^24^. From the view point of accuracy in the excitation energy, there are some other DFT formalisms such as the *GW* method^25^ and the time-dependent DFT^26^, but these methods require much higher calculation cost to construct a comprehensive spectral database. There are also other methods in dealing with the core-hole effect depending on the DFT formalism such as a method of Slater transition with half core hole in the Hartree-Fock-Slater method (or X *α*) method^27^ and approximation using an atom of the atomic number of *Z* + 1 including a full core hole^28^. The calculation condition adopted in this study is only one of them, and there is room for further discussion on how to incorporate this effect because the extent of the core-hole effect is affected by the life time of the core hole and its shielding effects which are largely dependent on the materials.

We carried out the calculations on 22,288 molecules which contain at least one carbon atom and no more than eight non-hydrogen atoms (C, O, N, and F) in QM9 dataset version 2^4,5^. Atomic configuration of each molecule was extracted from the dataset and was converted to the format of CASTEP input, namely “*.cell” files with a 15×15×15 Å periodic calculation cell. The Γ-point sampling was used for all calculations by explicitly setting KPOINT_MP_GRID, SPECTRAL_KPOINT_MP_GRID, and ELNES_KPOINT_MP_GRID as (1, 1, 1) in all the “*.cell” files. The atomic configurations in the dataset were used as is for all calculations, and only the electronic structure was optimized. For each molecule, symmetrically unique carbon sites were identified by a python program using the “PointGroupAnalyzer” class in the “pymatgen.symmetry” package, which is originally based on the spglib library^29^, and we adopted the parameter “tolerance” = 0.285. Calculations of spectrum were carried out considering the excitation at the symmetrically unique carbon sites. For molecules with multiple equivalent carbon sites, we only carried out calculation on only one of the equivalent sites to reduce calculation cost. For each symmetrically unique carbon site, three calculations for C-K edge spectrum and excitation energy were carried out as follows: First, excited electronic state with a full core hole in the 1 s orbital was calculated by the self-consistent field method. Secondly, theoretical C-K edge was calculated for the same system with consideration of a large number of unoccupied bands using the obtained electronic states in the first step. Finally, an electronic structure of the ground state was calculated for evaluating the theoretical excitation energy. The CASTEP code is based on the pseudopotential method and supports generation of a pseudopotential during runtime (“on-the-fly pseudopotential”). For all the three calculation steps, an on-the-fly ultrasoft pseudopotential was used for the symmetrically unique carbon site of interest in order to include the core hole effect and to evaluate excitation energies^16,18^. For the excited state, a full core hole in the 1 s orbital was introduced in an on-the-fly pseudopotential by setting “C:ex 2|1.4|6|11|11|20:21(qc = 7)1s1” in the “*.cell” file, and self-consistent field calculation was carried out to get total energy of the system and electron density. The C-K edge spectrum was then calculated using the calculation results on the excited state. The ground state was also calculated using an on-the-fly pseudopotential at the carbon site of interest but with the filled core state by setting “C:ex 2|1.4|6|11|11|20:21(qc = 7)1s2” in the “*.cell” file. The cutoff in number of unoccupied bands for the spectrum calculation was set to 1,000 by specifying “elnes_nextra_bands: 1000” in the “*.param” file. In each step, atomic energy of carbon at the site of interest was calculated by both pseudopotential method and all electron method and was stored in the “*.castep” file.

The spectral data is calculated through the dynamical structure factor for each eigen value of unoccupied states by calculating the transition matrix element using the projector augmented wave (PAW) approach^30^ within the dipole approximation^11^. The operator for transition process in ELNES and XANES can be written in the form of ${\widehat{O}}_{{\rm{E}}}={\rm{\exp }}(i{\bf{q}}\cdot {\bf{r}})$ and ${\widehat{O}}_{{\rm{X}}}={\rm{\exp }}(i(\omega /c)\widehat{{\bf{n}}}\cdot {\bf{r}})\widehat{\varepsilon }\cdot {\bf{p}}$, respectively, where **r** is the relative position of the excited electron from the core, **q** is the scattering vector, $\widehat{\varepsilon }$ is the polarization vector, $\widehat{{\bf{n}}}$ is the unit vector of propagation, and **p** is the momentum operator. In either case, the dipole approximation can be applied under $\delta \ll 1$: ${\rm{\exp }}(i\delta ) \sim 1+i\delta$. Thus, the transition matrix element is proportional to ${\phi }_{c}| {r}_{\alpha }| {\psi }_{n,k}$ for a specific positional operator ${r}_{\alpha }=x,y,z$, depending on direction of momentum transfer vector **q** in case of ELNES or that of propagation vector $\widehat{{\bf{n}}}$ in case of XANES, where *ϕ*_*c*_ and *ψ*_*n, k*_ are the core state and the unoccupied final state. In the CASTEP code, this is calculated through expansion with pseudofunctional basis set^11^ using PAW projector function ${\widetilde{p}}_{i}$, its corresponding pseudofunction ${\widetilde{\phi }}_{i}$, and a pseudofunction ${\widetilde{\psi }}_{n,k}$ corresponding to *ψ*_*n,k*_ as: ${\phi }_{c}|{r}_{\alpha }|{\psi }_{n,k}={\phi }_{c}|{r}_{\alpha }|{\widetilde{\psi }}_{n,k}+\sum _{i}({\phi }_{c}|{r}_{\alpha }|{\phi }_{i}-{\phi }_{c}|{r}_{\alpha }|{\widetilde{\phi }}_{i}){\widetilde{p}}_{i}|{\widetilde{\phi }}_{n,k}$. The dynamical structure factor then can be obtained by the square of the absolute value of the transition matrix element above, and spectrum is calculated for each positional operator *r*_*α*_ by merging the dynamical structure factors for each unoccupied eigen states. From physical point of view, the spectrum for a specific *r*_*α*_ is proportional to the spectrum taken by the incident direction of *r*_*α*_. The difference in spectra for *r*_*α*_ is originating from the anisotropic environment of the excitation site.

Some of the calculations of 133 molecules failed due to any errors in electronic structure optimization or other calculation procedures, and such molecules are not included in the dataset, resulting in the valid dataset for 22,155 molecules. For confirming the detailed calculation conditions, some of the raw CASTEP files (“*.cell”, “*.param”, and “*.castep”) are available at figshare^31^.

### Parsing data and creating database

#### Excitation energy

Since we adopted pseudopotential calculations using the CASTEP code, the excitation energy cannot be directly obtained from the difference of total energies. To obtain the excitation energy, we corrected the change in energies of the core orbitals using atomic energies obtained by all-electron and pseudo-atomic calculations using the reported procedure^14^. All the energies used for calculating the excitation energy were extracted from the output “*.castep” files. The atomic energies of the carbon atom obtained by all-electron and pseudo-atomic calculations are the same for all the carbon sites and are summarized in Table 1.

**Table 1:** Atomic energies used for correcting the excitation energies in the unit of eV.

State/Calculation	All electron	Pseudo atomic
Ground state	−1027.632	−148.5146
Excited state	−723.254	−241.7997

These energies were used for calculating the correction term $\Delta {E}_{{\rm{core}}({\rm{atom}})}=397.6631{\rm{eV}}$ for calculating the excitation energy ${E}_{{\rm{TE}}}=\Delta {E}_{{\rm{valence}}}+\Delta {E}_{{\rm{core}}({\rm{atom}})}$, where $\Delta {E}_{{\rm{valence}}}$ is given by the difference in the final energies of the ground state and the excited state.

#### Eigenvalues and dynamical structure factors

The CASTEP code outputs the energies and the dynamical structure factors for *r*_*α*_ = *x, y, z* to a text-formatted “*.elnes” file, and also outputs raw information about eigenvalue and transition matrix elements to “*.bands” and “*.eels_mat” files, respectively. For creating spectral database, we extracted data from “*.bands” and “*.eels_mat” files and formed spectra. The pairs of the eigenvalues and the transition matrix elements are parsed from “*.bands” and “*.eels_mat” file by a Python script which we published at GitHub^32^. The dynamical structure factors were obtained by the square of the absolute values of the transition matrix elements.

#### Site-specific C-K edge spectra

The C-K edge spectra for each symmetrically unique carbon site were calculated from the pairs of the eigenvalues and the dynamical structure factors by applying Gaussian smearing of 0.5 eV relative to the LUMO state eigenvalue. The total spectra were calculated by averaging over the three *r*_*α*_ directions.

#### Molecular C-K edge spectra

**Fig. 1:**
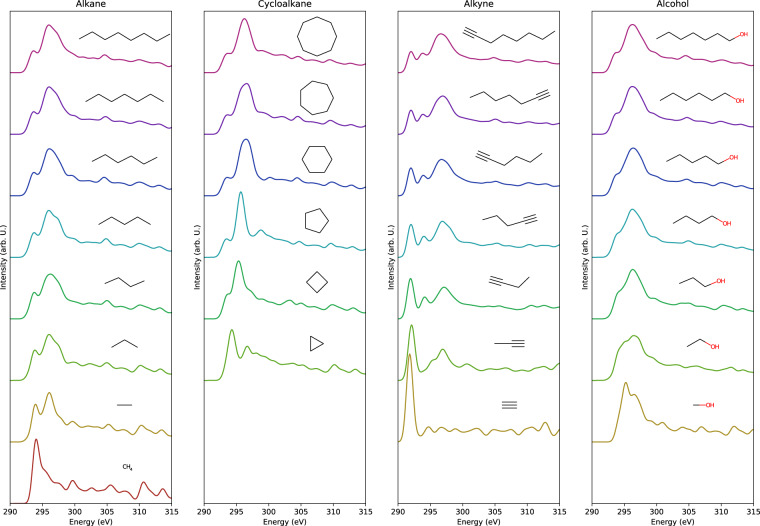
The calculated spectra smoothed with Gaussian filter with the standard deviation of 0.5 eV of some typical molecules in the dataset.

For each molecule, molecular spectra were obtained by shifting the site specific C-K edge spectra by the difference in excitation energies followed by averaging considering multiplicity of symmetrically unique sites. It should be emphasized that the relative energy difference between corresponding peaks in spectra from different sites is reported to reproduce that of experimental spectra^14^, which assures the summing up the calculated spectra from the individual sites to the calculate whole molecular spectra. Since there are some errors on the calculation of spectrum or excitation energy for any sites, C-K edge spectra of the molecules are missing. As a result, 22,155 molecular spectra were obtained in total from C-K edge spectra of 117,340 symmetrically unique sites. Figure 1 shows some typical spectra after applying smoothing with a Gaussian function of standard deviation of 0.5 eV.

## Data Records

The spectral data of the pair of raw eigenvalues and dynamical structure factors of each symmetrically unique carbon site are provided in HDF5 format at figshare^31^. For a typical use, the site and molecular specific C-K edge spectral data that are smoothed with a Gaussian filter with a standard deviation of 0.5 eV and sampled with 0.1 eV steps are provided as HDF5 files and csv files at figshare^31^. The site specific spectra database provides spectra of symmetrically unique carbon sites in terms of symmetrical equivalence along with their multiplicity and excitation energy. The molecular specific spectra database provides spectra of molecules obtained by weighted averaging considering multiplicity and excitation energy of the symmetrically unique sites in each molecule. For generating spectra with a desired smearing parameters, we also provide a Python script which can calculate smeared spectra from the HDF5 file of the site specific eigenvalues and dynamical structure factors. The raw CASTEP input and some output files are provided at figshare^31^ for reproduction and transparency of the calculation.

### Spectra database files in HDF5 format

The pairs of the eigenvalues and the dynamical structure factors, site specific spectra, and molecular spectra are separately provided in the format of HDF5 at figshare^31^.

#### Site specific eigenvalues and dynamical structure factors

This database contains the most primitive information related to spectrum: the final energies for the excited state and the ground state, the excitation energy, the site multiplicity, the eigenvalues and the dynamical structure factors, *i.e*. the square of the absolute values of the transition matrix element. The information of the *j*-th site of the *i*-th molecule is stored in the */i/j* group in the HDF5 file, where *i* is the ID of the molecule in the QM9 dataset and *j* is the integer number corresponding to the count of the sites in the xyz file of the QM9 dataset starting from 0. The eigenvalues and the dynamical structure factors are stored in data groups named */i/j/eigen_values* and */i/j/dsf*, respectively. The eigenvalues are stored as a one-dimensional array with length *n*_*band*_ in the unit of eV, where *n*_*band*_ is the number of the calculated bands. The dynamical structure factors were calculated as $| {\phi }_{c}| {r}_{\alpha }| {\psi }_{n,k}{| }^{2}$ in the unit of Å^2^ for the three *r*_*α*_(=*x, y, z*) and eigenstate index *n*, and thus stored as a two-dimensional array with shape (*n*_*band*_¸ 3). The number of electrons adopted in the calculation using pseudopotentials are stored as the attribute value “num_electrons” of each site group */i/j*. The site multiplicity, final energy of the ground state, final energy of the excited state, and excitation energy of the site are stored as the attribute values “multiplicity”, “final_energy_gs”, “final_energy_ex” and “excitation_energy” of the */i/j* group, respectively.

Note that the pairs of eigenvalues and dynamical structure factors below LUMO is also included. When forming a site-specific spectrum from these data, the dynamical structure factors below LUMO should be ignored, which can be achieved by ignoring the “num_electrons/2” levels from the lowest energy levels. In addition, for each eigenvalue, the dynamical scattering factor must be doubled because our calculation does not consider spin polarization. As for the site or molecular spectra, total non-directional spectra can be calculated as the average of the three *r*_*α*_(=*x, y, z*). In order to get a molecular spectrum, the site-specific spectra should be multiplied by the site multiplicity followed by summed up over the symmetrically unique sites.

#### Site specific spectra

The information of the *j*-th site of the *i*-th molecule is stored in the */i/j* group in the HDF5 file. Note that only one representative site is included among the symmetrically equivalent carbon sites. The spectral data is stored in datasets named */i/j/spectrum* and */i/j/energies*. The first, second, third, and fourth columns of */i/j/spectrum* are the total, and the *x*, *y*, and *z* direction spectra in the unit of Å^2^, respectively. */i/j/energies* is a one-dimensional array of the energy which is relative to the eigenvalue of the LUMO state in the unit of eV. The range of energies covers the minimum and maximum of energies relative to LUMO level with a margin of 5 eV. The site multiplicity, final energy of the ground state, final energy of the excited state, and excitation energy of the site are stored as the attribute values “multiplicity”, “final_energy_gs”, “final_energy_ex” and “excitation_energy” of the */i/j* group, respectively.

#### Molecular specific spectra

The information of *i*-th molecule is stored in the /*i* group in the HDF5 file. The spectral data is stored in a data set called */i/spectrum*, where the first, second, third, and fourth columns are the total, and the *x*, *y*, and *z* direction spectra in the unit of Å^2^, respectively. The energy corresponding to the */i/spectrum* in the unit of eV is stored in */i/energies*.

### Spectra database files in csv format

This CSV format database provides spectral data that are smoothed with a Gaussian filter with a standard deviation of 0.5 eV and thinned out in 0.1 eV steps. The data for each molecule is stored in a directory with a directory name corresponding to the molecule number *i* in QM9. In each directory, three types of data are stored in CSV file format: spectral data of the molecule, site-specific spectral data, and data of the molecular structure. The file name of the molecular spectrum data is “*i*.csv”, the file name of the site spectrum data is “*i*_*j*_*m*.csv”, and the file name of the molecular structure data is “*i*_structure.csv”, where *i* is the molecule number in the QM9 database, *j* is the site number in molecule *i*, and *m* is the multiplicity of site *j* in molecule *i*. The file format of the csv file for spectral data and structures are summarized in Tables 2 and 3.

**Table 2:** CSV file format for molecular and site-specific C-K edge core-loss spectra.

Column	Header	Content	Unit
1	energy	energy loss	eV
2	tot	total dynamical structure factor	Å^2^
3	x	*x* component of dynamical structure factor	Å^2^
4	y	*y* component of dynamical structure factor	Å^2^
5	z	*z* component of dynamical structure factor	Å^2^

**Table 3:** CSV file format for a molecular structure.

Column	Header	Content	Unit
1	site	site number	
2	specie	atomic specie	
3	x	*x* coordinate of the site	Å
4	y	*y* coordinate of the site	Å
5	z	*z* coordinate of the site	Å
6	multiplicity	multipliticy of the site	
7	representative	representative site used for calculation	

### Raw CASTEP files

The most of raw CASTEP input and output files for carbon sites are available at figshare^31^ except for PBE pseudopotential files and spectra data files. This record covers input files which are necessary for recalculation and output files which are sufficient for confirming calculation process.

## Technical Validation

### Comparison to experimental spectra

Some of the calculated spectra were compared with experimental spectra. We extracted experimental spectral data of 44 molecules from the Gas Phase Core Excitation Database published and maintained by the Hitchcock group^33^, and compared their energy position and shape of the spectrum with those of our calculated spectral data. The list of the 44 molecules used for comparison is summarized in Table 4.

**Table 4:** List of the ID in QM9 dataset, molecule name, and reference to the original article of the 44 molecules extracted from the Gas Phase Core Excitation Database published and maintained by the Hitchcock group^33^ for the comparison of experimental spectral data to our calculated spectral data.

ID in QM9	name of molecule^ref. no.^
#4	Acetylene^40^
#7	Ethane^41^
#10	Acetonitrile^42^
#11	Acetaldehyde^43^
#13	*n*-Propane^44^
#16	Cyclopropane^45^
#21	Isobutane^44^
#29	2-Butyne^46^
#33	Propargyl alcohol^47^
#37	Methyl formate^47^
#39	*n*-Butane^48^
#40	*n*-Propanol^47^
#47	Cyclobutane^45^
#51	Imidazole^49^
#54	2,2-Dimethylpropane^44^
#55	*t*-Butyl alcohol^50^
#83	Isopentane (i.e. 2-Methylbutane)^44^
#133	*n*-Pentane^44^
#136	Diethyl ether^51^
#156	Cyclopentene^45^
#159	Cyclopentane^45^
#160	Tetrahydrofuran^52^
#214	Benzene^53^
#215	Pyridine^54^
#218	*s*-Triazine^49^
#266	Ethyl carbamate^51^
#286	Alanine^55^
#543	*n*-Hexane^44^
#659	Tetrahydropyran^52^
#940	Aniline^56^
#949	Phenol^57^
#1132	Isopropyl ether^51^
#2071	Cyclohexanone^58^
#4208	Monofluorobenzene^53^
#4336	*m*-Xylene^59^
#4563	*p*-Xylene^59^
#4591	Hydroquinone^57^
#4958	*o*-Xylene^59^
#5357	Benzaldehyde^60^
#7723	1,3-Cyclohexanedione^58^
#10793	1,4-Cyclohexanedione^58^
#12304	1,2-Cyclohexanedione^58^
#17954	Cyclooctatetraene^45^
#23866	Hexafluoroethane^46^

The superscript numbers to the name of the molecules are the reference numbers of the original literatures of the spectra used for the comparison.

**Fig. 2:**
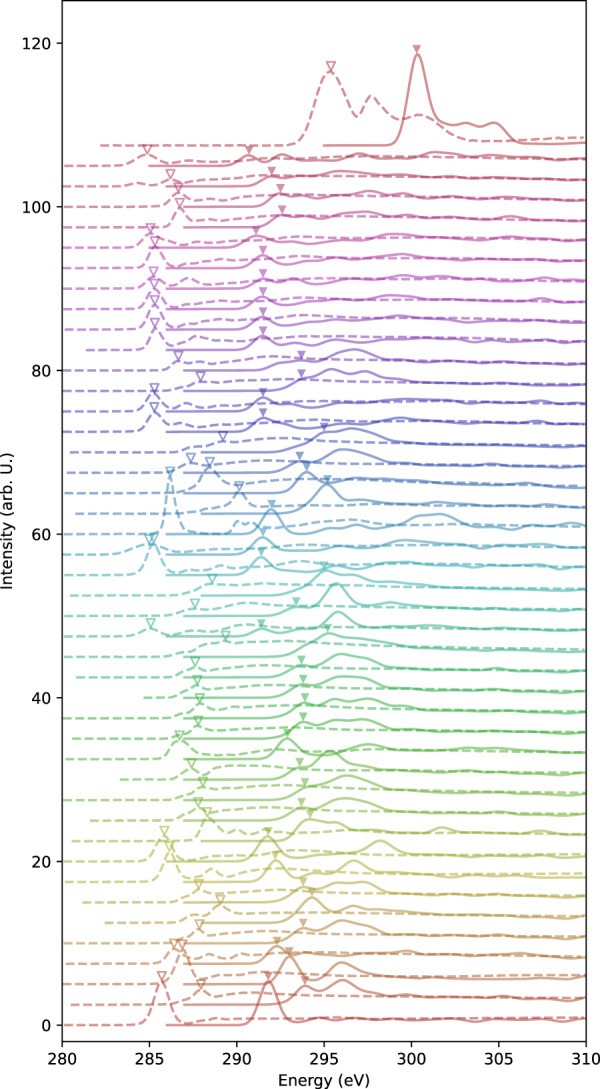
Comparison of the shape of the spectra between the calculated spectra smoothed with Gaussian filter with the standard deviation of 0.5 eV (solid lines) and the 44 experimental spectra extracted from literatures summarized in Table 4 (dotted lines). The triangle markers denote the position of the first peak. Spectra are shifted for visibility, and the order of the spectra from bottom to top corresponds to the order of molecules listed in Table 4.

**Fig. 3:**
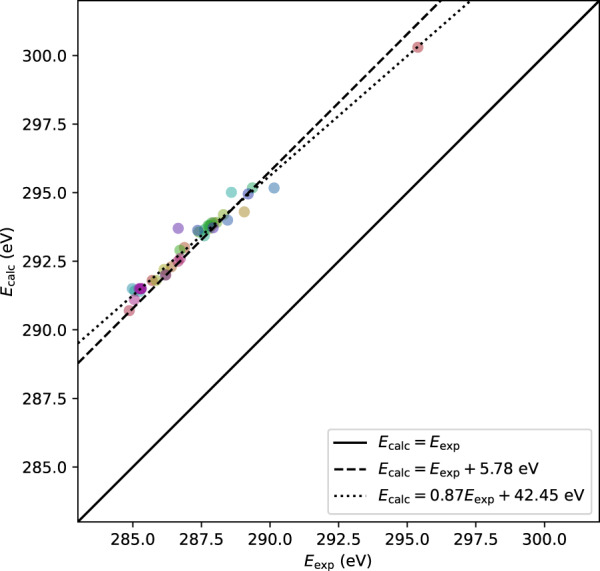
Comparison in the energy position of first peak between the calculated spectra and experimental spectra in literatures, *E*_*calc*_ and *E*_*exp*_, respectively. Solid line shows the line: *E*_*calc*_ = *E*_*exp*_. Dashed line shows the line only considering energy shift: ${E}_{{\rm{calc}}}={E}_{{\rm{\exp }}}+5.78{\rm{eV}}$. Dotted line shows the line obtained by linear regression: ${E}_{{\rm{calc}}}=0.87{E}_{{\rm{\exp }}}+42.45{\rm{eV}}$. The colors of markers are the same for the line color in Figs. 2 and 4.

Figure 2 shows the calculated spectra after applying smoothing with a Gaussian function of standard deviation of 0.5 eV and experimental spectra. A mismatch in the absolute value of transition energy can be seen between the experimental and calculated spectra. To analyze the difference in the transition energy, we extracted the positions of the first peak. Figure 3 shows a scatter plot of the energy position of the first peak in the experimental data and our calculations. The data points are distributed linearly, which confirms that it is a systematic misalignment. We tried linear regression and obtained a fitting line of ${E}_{{\rm{calc}}}=0.87{E}_{{\rm{\exp }}}+42.45{\rm{eV}}$. It was also found that taking into account an energy shift of 5.78 eV, assuming the slope of 1.0, is a sufficient approximation. Practically, this means that energies of our calculation tends to be higher by 5.78 eV than the experiment in terms of energy. These systematic deviations indicate that the calculated transition energy are of sufficient quality to withstand comparison with that of experimental data by considering the systematic error of 5.78 eV. It should be noted that this level of systematic errors in the transition energies of core-loss spectrum calculations is generally observed in various calculation methods^16,18,34–38^.

**Fig. 4:**
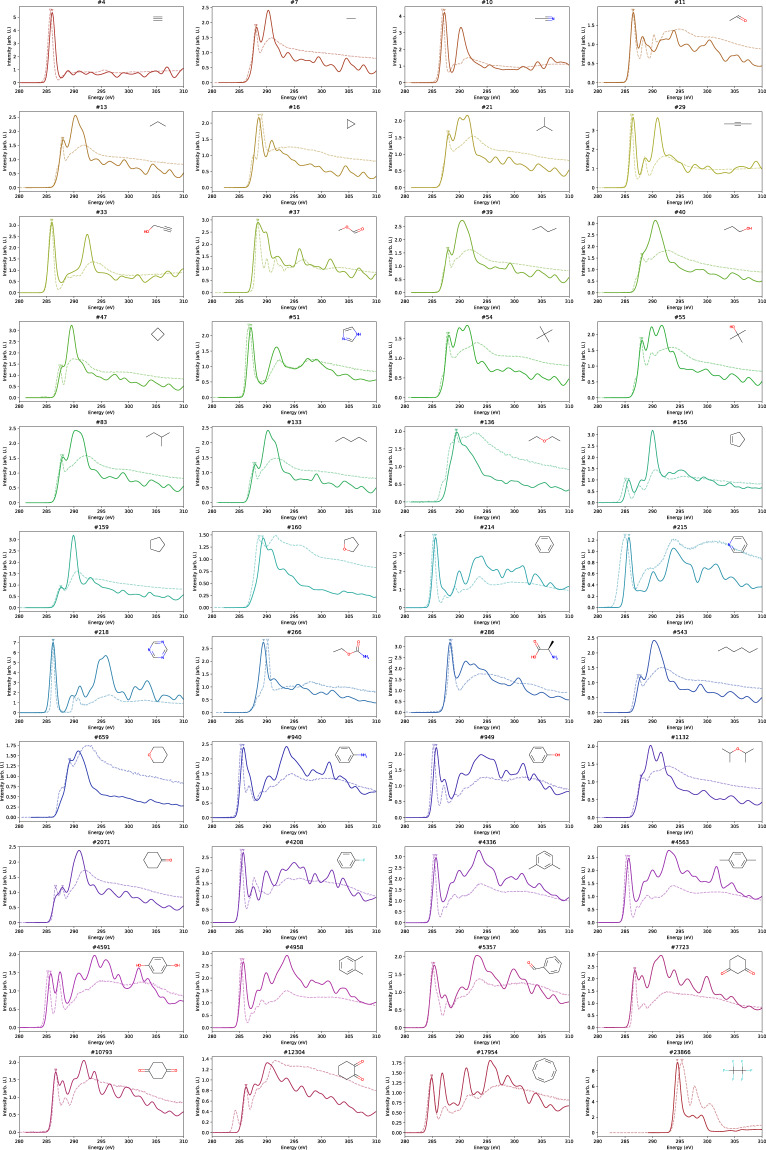
Comparison of the shape of the spectra between the calculated spectra smoothed with Gaussian filter with the standard deviation of 0.5 eV and shifted by 5.78 eV (solid lines) and experimental spectra in literatures (dotted lines) for each molecule.

For comparison of the spectral features, the experimental spectrum and our calculated spectrum with the energy position shifted by 5.78 eV are shown in Fig. 4. There are a few molecules for which the spectral shapes do not match, but the agreement is generally good, taking into account the existence of the variation in the experimental spectral data depending on the measurement conditions. Those spectral features can satisfactorily reproduce the experimental spectra, and the chemical shift between those peaks are quantitatively reproduced by the present method. It is worthwhile that the chemical shift among different molecules can be quantitatively reproduced by the present calculation.

The simulated spectral intensities tend to become weaker than that of the experiment in high energy regions above about 310 eV, which is attributed to the fact that the number of unoccupied levels is only considered up to 1,000 and only intra-atomic transitions are taken into account.

Concerning the effect of spin polarization, we also carried out calculations considering spin polarization for the 44 molecules, and found only a small difference in excitation energy less than 0.41 eV compared to that of non-magnetic calculation, which indicates the consideration of spin does not yield significant difference compared to the mismatch between simulated excitation energies and experimental ones.

### Calculation cell size dependence

**Fig. 5:**
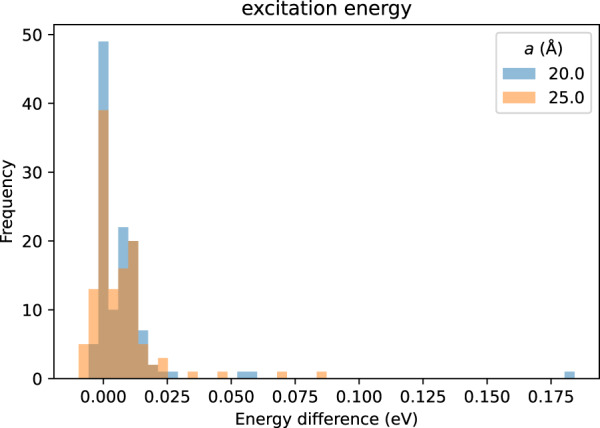
Histogram of excitation energy difference for different cell sizes compared to that of the 15 Å cubic cell.

We checked calculation cell size dependence of the excitation energies for the 44 molecules in Table 4 from 15.0 to 25.0 Å cubic cell. Figure 5 shows the histograms of the deviations of excitation energies compared to those of 15.0 Å cubic cell except for the site 4 of #5357 whose calculations did not converged for the larger cell sizes. The largest deviation of the excitation energy was 184 meV, but most of them are located within 25 meV, which is relatively small compared to the realistic smearing width of the spectrum like 0.3 eV, and they do not have large impact when considered as data for comparison with experiments or for machine learning.

## Usage Notes

In this work, we present raw files of CASTEP, which are of use for confirming the detail of calculation conditions. The spectra database files contain all the calculated spectra, and can be used for finger-print method and spectrum informatics. Spin polarization and spin-orbit interaction are not considered for all calculations. The spectra was calculated using 1,000 unoccupied states and only inner atom transition was considered, which yields mismatch to experimental results especially in high energy loss regions. The absolute value of the excitation energies tends to be overestimated systematically compared to the experimental values by about 6 eV as can be seen in Fig. 3, which might be due to the used of difference in atomic energies between ground states and excited states for the correction of energies calculated using pseudopotentials.

## Data Availability

A proprietary code, Academic Release CASTEP version 8.0^6–11^ was used to perform DFT and core-loss spectra calculations. The configuration files used in the calculation is provided for reproducing the site C-K edge spectra at figshare^31^ along with some of the output files for confirmation of calculation condition. For making input files, parsing output files, creating database and visualization, we have used the following python libraries: Numpy, Pandas, h5py, rdkit and Matplotlib. The Python code used for parsing the CASTEP input and output files is available at GitHub^32^ under the MIT license. The Python code used for making the Gaussian smeared spectra dataset from the database HDF5 file of eigenvalues and dynamical structure factors is also available at GitHub^39^ under the MIT license, and can be used for making spectra with arbitrary smearing parameters.
